# Can accelerated ageing models inform us on age‐related tauopathies?

**DOI:** 10.1111/acel.13830

**Published:** 2023-04-03

**Authors:** Zhuang Zhuang Han, Alex Fleet, Delphine Larrieu

**Affiliations:** ^1^ Department of Pharmacology University of Cambridge Tennis Ct Rd Cambridge CB2 1PD UK

**Keywords:** ageing, animal models, progeria, tau, tauopathy

## Abstract

Ageing is the greatest risk factor of late‐onset neurodegenerative diseases. In the realm of sporadic tauopathies, modelling the process of biological ageing in experimental animals forms the foundation of searching for the molecular origin of pathogenic tau and developing potential therapeutic interventions. Although prior research into transgenic tau models offers valuable lessons for studying how tau mutations and overexpression can drive tau pathologies, the underlying mechanisms by which ageing leads to abnormal tau accumulation remains poorly understood. Mutations associated with human progeroid syndromes have been proposed to be able to mimic an aged environment in animal models. Here, we summarise recent attempts in modelling ageing in relation to tauopathies using animal models that carry mutations associated with human progeroid syndromes, or genetic elements unrelated to human progeroid syndromes, or have exceptional natural lifespans, or a remarkable resistance to ageing‐related disorders.

## Graphical abstract

Modelling the process of biological ageing in experimental animals is necessary for understanding how ageing leads to abnormal tau accumulation and tau‐related neurodegeneration. As discussed in this review, recent attempts to model ageing in relation to tauopathies have utilised animal models with mutations associated with human progeroid syndromes, or with exceptional natural lifespans, or resistance to age‐related disorders.

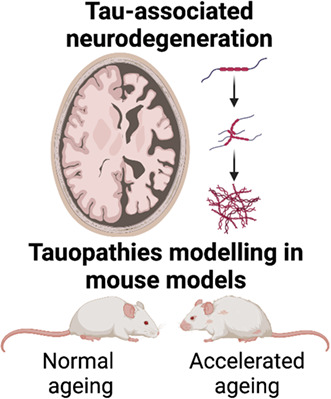

**Abbreviations**
AD: Alzheimer's disease
AGD: argyrophilic grain disease
A‐T: ataxia telangiectasia
ATM: ataxia‐telangiectasia mutated serine/threonine kinase
CBD: corticobasal degeneration
CS: Cockayne syndrome
DSBs: double‐stranded DNA breaks
FTLD: frontotemporal dementia
HGPS: Hutchinson Gilford progeria syndrome
KL: Klotho
MAPs: microtubule‐associated proteins
MAPT: microtubule‐associated protein tau
NCDs: non‐communicable conditions
NMR: naked mole rat
PiD: Pick's disease
PS: progeroid syndromes
PSP: progressive supranuclear palsy
SAMP: senescence‐accelerated prone mouse
WS: Werner syndrome
XP: xeroderma pigmentosum

## 1 INTRODUCTION

Ageing is a time‐dependent progressive decline of physiological functions that compromises the ability to adapt to metabolic stresses. As a main risk factor for many prevalent non‐communicable conditions (NCDs), advancing age not only contributes to life‐threatening diseases (e.g., cancer [White et al., 2014], cardiovascular diseases [Rodgers et al., 2019] and neurodegeneration [Hou et al., 2019]), but also to less lethal conditions that significantly compromise life quality (e.g., cataract [Nirmalan et al., 2004], hypertension [Buford, 2016] and diabetes [Khan et al., 2020]). In the past few decades, human life expectancy has greatly increased worldwide, and it is expected to continue to increase (Bell & Miller, 2005; United Nations—Department of Economic and Social Affairs, 2019). While this is linked to a spectacular advancement in modern medical technologies, a longer life expectancy also confers a greater lifetime exposure to internal and external risk factors that may cumulatively result in ailments. Hence, compressing age‐related morbidity and further extending the human healthspan require substantial breakthroughs to understand the biology of ageing at an organismal, organ, cellular and molecular level.

One major drawback in in vivo ageing research is the duration and the cost of the experiments due to the requirement of keeping the animals for most of their lifespan (over 2 years for a typical mouse model) to be able to study the ageing process. Fuelled by the discovery of ageing‐related features in a variety of accelerated ageing (progeroid) syndromes, the past two decades has seen both advancement in understanding the underlying molecular mechanism behind these rare diseases and remarkable efforts in utilising progeroid pathological ageing to unveil the mystery of physiological ageing and ageing‐related disorders. In the realm of neurodegenerative diseases, some strides in understanding have been helped by the analysis of human patient samples. But advances in this period have also arisen from the emergence of new animal/tissue culture models that age faster while recapitulating key biochemical ageing‐related features such as senescence, nuclear envelope impairment and elevated levels of genomic damage—to name a few. Here, we recap recent attempts in utilising model organisms, which exhibit an accelerated or decelerated rate of ageing, to study neurodegenerative disorders that are predominantly driven by pathological changes of the tau protein (tauopathies). To understand the advantages and disadvantages of using fast/slow ageing models, it is necessary to briefly consider the ageing‐dependent nature of tauopathies and widely used animal models in tauopathy research.

### 1.1 Microtubule‐associated protein tau and tauopathies

Tau proteins are a group of low molecular weight microtubule‐associated proteins, of which there are six isoforms produced by alternative splicing of the *MAPT* gene, located on chromosome 17q21 (Goedert et al., 1988, 1989). A highly conserved protein, tau is expressed across many animal species, including human, mouse, rat and bovine species (Adolf, 1989; Takuma et al., 2003). Transcripts of tau are found primarily, though not exclusively, within the central nervous system, particularly in neurons. As would be expected of microtubule‐associated proteins (MAPs), the primary physiological function of tau is binding to microtubules, stabilising and mediating their assembly and modulating vesicle/organelle transport along microtubules (Binder et al., 1985; Weingarten et al., 1975). Within neurons, tau is primarily located within axons, though may also be found within somatodendritic compartments such as the cell membrane (Arrasate et al., 2000), mitochondria (Li et al., 2016) and nucleus (Wang et al., 1993). In addition, cellular localisation of tau allows it to participate in other vital cellular activities, such as regulating axonal transport (Morris et al., 2021), protecting against genetic damage (Sultan et al., 2011; Violet et al., 2014), modulating synaptic plasticity (Wang, Khandelwal, et al., 2022) and assisting in neuronal maturation (Fiock et al., 2020).

The term ‘tauopathy’ refers to a group of neurodegenerative diseases pathologically characterised by the accumulation of insoluble intracellular tau inclusions in the central nervous system, with symptoms of dementia and/or parkinsonism (Kovacs, 2015). Some examples of tauopathy include Alzheimer's disease (AD), argyrophilic grain disease (AGD), globular glial tauopathy, progressive supranuclear palsy (PSP), Pick's disease (PiD) and corticobasal degeneration (CBD; Review by [Götz et al., 2019]). Based upon the primary contributor of pathology, tauopathies can be classified as primary tauopathies, in which tau aggregation plays a prominent role in disease pathogenesis, and secondary tauopathies, where disease progression is mainly propelled by abnormalities of other proteins. Based upon its aetiology, tauopathies can be divided into two subtypes: familial and sporadic. Familial tauopathies are caused by mutations in the *MAPT* gene and are often autosomal dominant. To date, more than 100 tau mutations have been documented yet some do not have known functions in pathogenesis (ALZFORUM – Mutation Database, n.d.). Most exonic pathogenic mutations lie in the coding regions of exons 9, 10, 11 and 12, corresponding to the repeated microtubule‐binding domain of tau. Intronic mutations affect the ratio of 3R:4R tau splicing isoforms. Familial tauopathies generally associate with early onset of symptoms (~ 58.5 years in FTLD‐MAPT [Moore et al., 2020]). On the other hand, sporadic tauopathies are not hereditary and have delayed onset of symptoms (>65 years in most sporadic AD cases [Rabinovici, 2019]). Not linked to tau genetic mutations or any specific environmental factors, sporadic tauopathies occur in a seemingly unpredictable manner, suggesting a complex and multifactorial nature of its pathogenesis.

**FIGURE 1:**
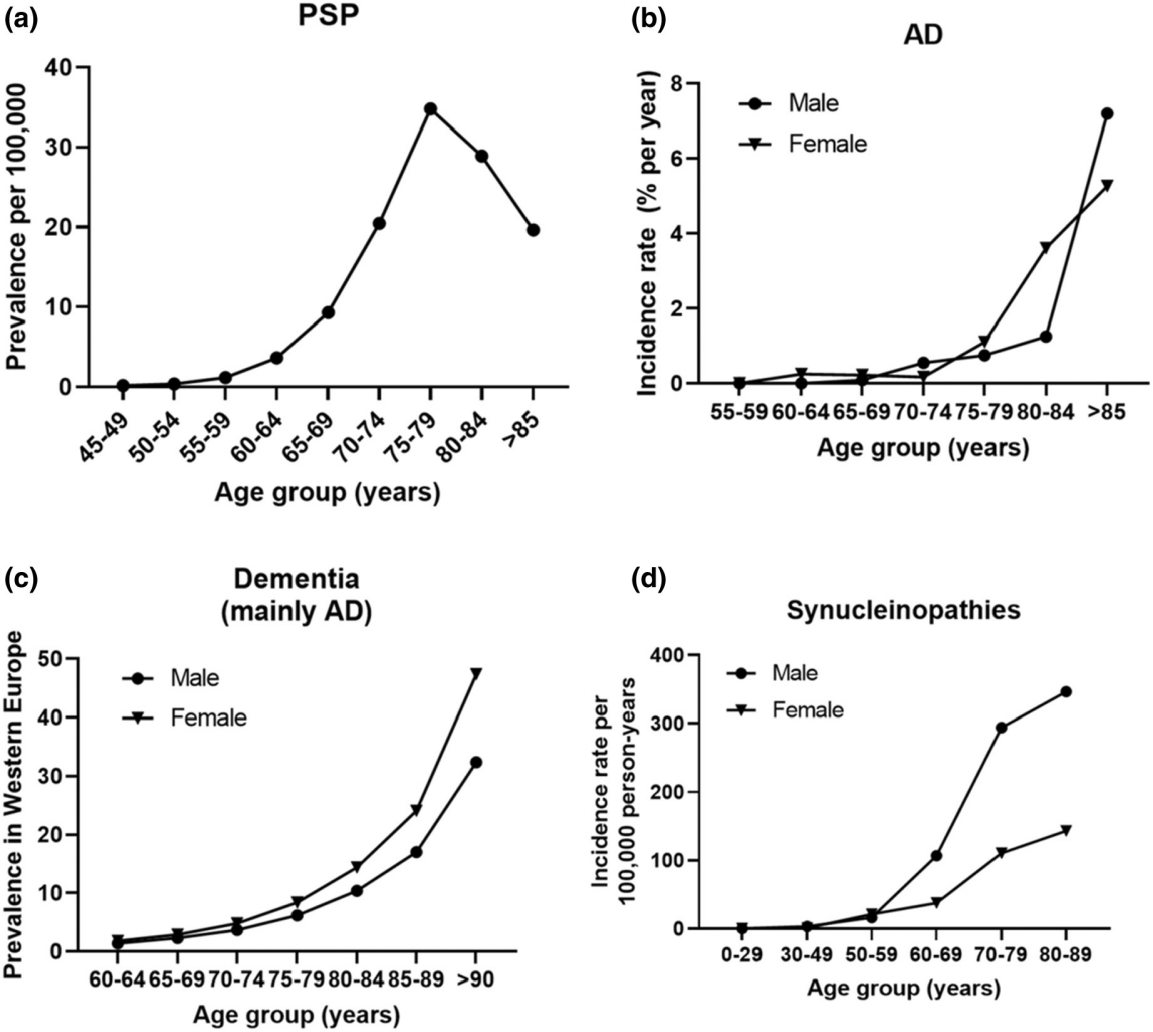
Correlation of age and prevalence/incidence of neurodegenerative diseases—progressive supranuclear palsy (a), Alzheimer's disease (b), general dementia (c) and synucleinopathies (d). Plots were created from data from Viscidi et al. (2021), Kawas et al. (2000), Prince et al. (2014), and Savica et al. (2013).

Among all known risk factors for sporadic late‐onset tauopathies, chronological age is strongly correlated with the prevalence of the disease. For instance, a recent epidemiological analysis showed a positive correlation between age and prevalence of progressive supranuclear palsy, a primary tauopathy (Viscidi et al., 2021; Figure 1a). The same correlation has also been established in Alzheimer's disease, a secondary tauopathy. An early longitudinal study conducted in the United States demonstrated a strong positive correlation between age and the incidence rate of Alzheimer's disease in both male and female participants (Kawas et al., 2000; Figure 1b). In 2014, a meta‐analysis of the prevalence of dementia (62% of which was Alzheimer's disease) in Western Europe illustrated the same correlation (Prince et al., 2014; Figure 1c). Notably, ageing is also an important risk factor in neurodegenerative diseases in which pathogenesis is driven by proteins other than tau. For example, advanced age also appears to correlate with high incidence of parkinsonism (Figure 1d), a neurological condition primarily caused by defects in α‐synuclein (Savica et al., 2013). The sex bias in dementia related to tau or synuclein may be explained by sex hormones (Rajsombath et al., 2019; Sundermann et al., 2020; Yang et al., 2022), or sex‐related differences in brain connectome and specific genes/transcription factors (López‐Cerdán et al., 2022; Shokouhi et al., 2020).

### 1.2 Tauopathy rodent models

Model organisms form the foundations of sporadic tauopathy research, as they offer physiologically relevant platforms to study the influences of genetic, nutritional and environmental factors, and observe pathological changes at different time points of disease progression. Importantly, using model organisms with short natural lifespan allows for investigation of late‐onset disorders in a timely manner. In the realm of tauopathy research, more than 40 animal models have been developed over the past two decades (ALZFORUM—Animal Models, n.d.). The summary of single knock‐in transgenic tau rodent models captured in Table 1 highlights two limitations in these models. First, most of these animal models frequently rely on an overexpression of human tau protein (huTau) with disease‐related mutations, for example, P301L (Lewis et al., 2000) and R406W (Frost et al., 2016) in FTLD‐MAPT (frontotemporal lobar degeneration associated with *MAPT* mutations). In consequence, they recapitulate processes occurring in genetic tauopathies, but not necessarily the events of sporadic tauopathies. Second, overexpression of mutant tau significantly accelerates pathogenesis and therefore does not necessarily reflect the ageing‐dependent nature of idiopathic tauopathies. Indeed, only two out of 23 rodent models in Table 1 have a late‐onset cognitive impairment (>12 months in an average lifespan of 2 years for laboratory mice (Dutta & Sengupta, 2016) and 3 years for laboratory rats [Quinn, 2005; Sengupta, 2013]). As mimicking an aged cellular/physiological environment is an important practical and theoretical consideration that facilitates research into the origin of sporadic tauopathies, it is necessary to seek ways to combine the knowledge we inherited from studies using TgTau animal models and the lessons we learned from accelerated/decelerated ageing animal models.

**TABLE 1:** Transgenic single knock‐in tau rodent models developed in the last two decades (ALZFORUM—Animal Models, n.d.).

Organism	Model	Transgene	Cognitive impairment onset (m.)	Promoter	Established in
Mouse	hTau (Andorfer et al., 2003)	HuTau	6	Tau promoter	2003
	hTau‐A152T (Maeda et al., 2016)	HuTau (1N4R, A152T)	10	CaMKIIα promoter	2016
	hTau.P301S (Allen et al., 2002)	HuTau (0N4R, P301S)	3	Thy‐1 promoter	2002
	JNPL3(P301L) (Lewis et al., 2000)	HuTau (0N4R, P301L)	Unknown	Mouse prion promoter	2000
	MAPT knock‐in (Saito et al., 2019)	MuTau replaced with HuTau	Unknown	Mouse *mapt* promoter	2019
	mThy‐1 3R tau (line 13) (Rockenstein et al., 2015)	HuTau (3R, L226V, G272V)	8‐10	Mouse Thy‐1 promoter	2015
	rTgTauEC (de Calignon et al., 2012)	HuTau (4R0N, P301L)	16	Neuropsin promoter	2012
	rTg(tauP301L)4510 (Santacruz et al., 2005)	HuTau (4R0N, P301L)	2.5	tTA promoter	2005
	RW Tg mice (Zhang et al., 2004)	HuTau (4R2N, R406W)	Unknown	Mouse prion promoter	2004
	Tau264 (Umeda et al., 2013)	HuTau (3R and 4R)	6	CaMKIIα promoter	2013
	Tau35 (Bondulich et al., 2016)	HuTau (187‐441 a.a.)	8	Human tau promoter	2016
	Tau4RTg2652 (Wheeler et al., 2015)	HuTau (4R1N, WT)	3	Thy1 promoter	2015
	Tau609 (Tau 10+16) (Umeda et al., 2013)	HuTau (4R2N, IVS10+16 C>T)	6	CaMKIIα promoter	2013
	TauC3 (Kim et al., 2016)	HuTau (0N4R, Δ20)	1.3	BAI1‐AP4 promoter	2016
	Tau Exon 10 KO (Gumucio et al., 2013)	Deletion of exon 10 in muTau	Unknown	N/A	2013
	TauΔK280 (Eckermann et al., 2007)	HuTau (4R2N, ΔK280)	16	CaMKIIα promoter	2007
	Tau P301L (Terwel et al., 2005)	HuTau (4R2N, P301L)	5	Mouse Thy1 promoter	2005
	Tau P301S (PS19) (Yoshiyama et al., 2007)	HuTau (4R1N, P301S)	6	Mouse prion promoter	2007
	Tau R406W (Tatebayashi et al., 2002)	HuTau (4R2N, R406W)	16	CaMKIIα promoter	2002
	TauRDΔK280 (Mocanu et al., 2008)	HuTau (244‐372 a.a.)	10	CaMKIIα promoter	2008
	Tau V337M (Tanemura et al., 2001)	HuTau (4R2N, V337M)	11	PDGF‐β promoter	2001
	THY‐Tau22 (Schindowski et al., 2006)	HuTau (4R2N, G272V, P301S)	6	Thy1 promoter	2006
	TMHT (Flunkert et al., 2013)	HuTau (4R2N, V337M, R406W)	5	Thy1 promoter	2013
Rat	SHR24 (Filipcik et al., 2012)	HuTau (4R2N, 151‐274 a.a. and 306‐391 a.a.)	Unknown	Mouse Thy1 promoter	2012
	SHR318 (Zilka et al., 2006)	HuTau (4R2N, 151‐391 a.a.)	4.5	Mouse Thy1 promoter	2006
	SHR72 (Koson et al., 2008)	HuTau (4R2N, 151‐391 a.a.)	Unknown	Mouse Thy1 promoter	2008

## 2 AGEING MODEL ORGANISMS IN NEURODEGENERATION RESEARCH

**FIGURE 2:**
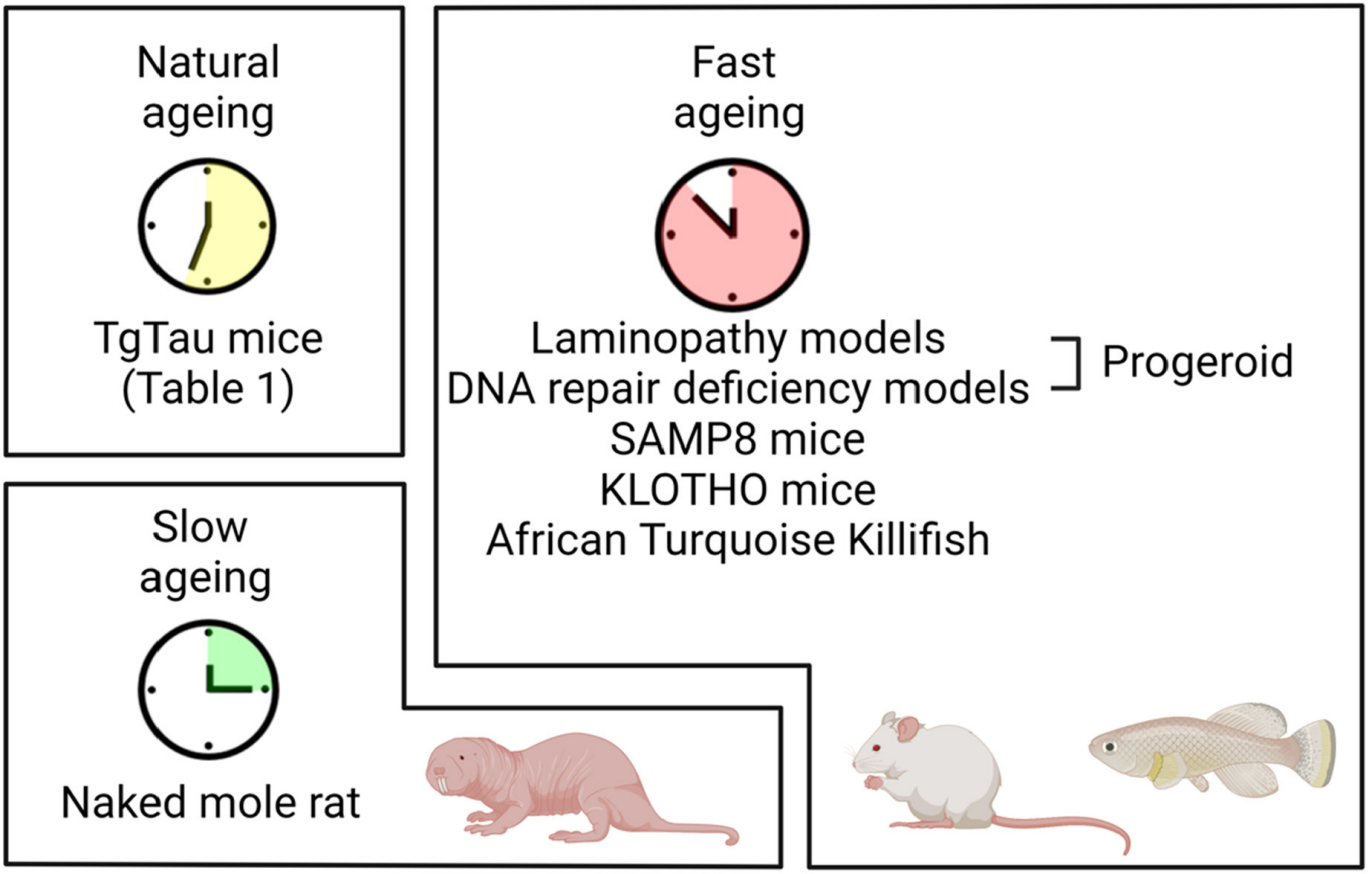
Ageing model organisms used in tauopathy research. Created with BioRender.com.

In search for avenues to combat ageing and age‐related morbidities, two types of ageing processes have been studied: inborn normal (chronological) ageing and premature pathological ageing. Normal physiological ageing is associated with various defects at the subcellular level, including genome instability (Vijg & Suh, 2013), telomere shortening (Aubert & Lansdorp, 2008), mitochondrial and metabolic dysfunction (Trifunovic & Larsson, 2008), alterations in epigenetics (Saldanha & Watanabe, 2015), altered intercellular communication (Salminen et al., 2012), stem cell exhaustion (Oh et al., 2014), cellular senescence (di Micco et al., 2021), deregulated nutrient sensing (Houtkooper et al., 2010), loss of proteostasis (Santra et al., 2019), disabled macroautophagy (Pyo et al., 2013), chronic inflammation (Chung et al., 2019) and dysbiosis (Haran & McCormick, 2021), collectively known as ‘the 12 hallmarks of ageing’ proposed by Lopez‐Otin and colleagues (López‐Otín et al., 2023). As shown in Figure 1, in the following section, we will summarise two biological extremes: accelerated ageing models and decelerated ageing models. These have helped shed light on the basic genetic and physiological mechanisms associated with natural ageing and age‐dependent diseases (Figure 2).

### 2.1 Accelerated ageing models in tauopathy research

In the field of cellular ageing and neurodegeneration, approaches to study the effects of ageing include the following: first, use of genetically modified animals that express mutated proteins which have been associated with human premature ageing syndromes (also called progeroid syndromes‐ PS). PS are rare congenital/genetic disorders that recapitulate some pathological features of normal ageing in an accelerated manner and thus provide potential insights into the natural ageing process. Most human PS are caused by either defects in the nuclear lamina, hence, the name ‘laminopathies’, or deficiencies in the DNA repair machineries. Table 2 offers a non‐exclusive summary of human PS. There have been attempts to exploit defects in these two cellular processes to mimic ageing in tauopathy model organisms; their benefits and limitations are summarised in Table 3. Second, the use of natural genetic variants that occur in animal lines that display phenotypes of premature ageing—these would include animals harbouring the polygenic SAMP8 trait or being homozygous for null alleles of the Klotho gene (Akiguchi et al., 2017; Kuro‐o et al., 1997). Third, using vertebrate organisms that have a naturally short lifespan, such as the African Turquoise Killifish, proved to be an effective model to study ageing.

**TABLE 2:** Examples of genetic progeroid syndromes and associated neurological phenotypes.

Progeroid syndromes	Dominant/recessive	Mutated gene(s)	Affected protein(s)	Affected cellular mechanism	Neurological phenotypes
Ataxia telangiectasia (Rothblum‐Oviatt et al., 2016)	Recessive	*ATM*	ATM (Ataxia Telangiectasia, Mutated)	DNA damage response	Motor neuron defects, progressive cerebellar ataxia
Cockayne syndrome (Laugel, 2013)	Recessive	*ERCC8; ERCC6*	CSA, CSB	Nucleotide excision repair	Mental retardation, microcephaly, retinal atrophy and progressive neurodegeneration
Hutchinson‐Gilford progeria syndrome (Pollex & Hegele, 2004; Ullrich & Gordon, 2015)	Dominant	*LMNA*	Lamin A	Nuclear lamina	None
Nestor‐Guillermo progeria syndrome (Cabanillas et al., 2011)	Recessive	*BANF1*	Barrier‐to‐autointegration factor	Nuclear lamina	None
Restrictive dermopathy (Pradeep et al., 2022)	Recessive	*ZMPSTE24*	Lamin A	Nuclear lamina	None
Werner syndrome (Muftuoglu et al., 2008)	Recessive	*WRN*	WRN helicase	RecQ protein‐like helicase	Non‐AD senile dementia, schizophrenia, cerebrovascular disease and peripheral neuropathy
Xeroderma pigmentosum (Lehmann et al., 2011)	Recessive	*DDB2; ERCC2; ERCC3; ERCC4; CC5; XPA; XPC*	XP DNA repair proteins	Nucleotide excision repair	Subtype‐dependent (see Table 3)

**TABLE 3:** Benefits and limitations of specific progeric models for tauopathy research.

	Progeroid syndromes	Models	Benefits	Limitations
Laminopathy models	Hutchinson–Gilford progeria syndrome	Progerin knock‐in mice (Jung et al., 2012; Osorio et al., 2011)	N/A	Progerin absent from neuronal and glial cells No neurological phenotype Tau‐specific pathology has not been reported
		Inducible progerin transgenic mice (Baek et al., 2015)	Progerin specifically expressed in neurons Severe nuclear distortion	No neurological manifestation or behavioural changes Tau‐specific pathology has not been reported
		Human iPSC‐derived neuron expressing progerin (Miller et al., 2013)	Recapitulate key molecular markers of ageing and Parkinson's disease	Do not necessarily reflect a physiologically relevant environment
	Restrictive dermopathy	ZMPSTE24‐deficient mice (de Carlos et al., 2008; Yang et al., 2015)	Neurological abnormalities Investigates lamin A processing in a progerin‐independent manner	Tau‐specific pathology has not been reported
DNA repair deficiency models	Cockayne syndrome	Csa^−/−^ mice (Jaarsma et al., 2011)	Minor phenotypes in the central nervous system	No detectable neurodegeneration Tau‐specific pathology has not been reported
		Csa^−/−^/Xpa^−/−^ mice (Jaarsma et al., 2011)	CS‐related neurological dysfunction	Double mutation adds another layer of complexity Tau‐specific pathology has not been reported
		Csb^R571X/R571X^ rat (Xu et al., 2019)	Multiple neurological defects: cerebellar atrophy, hippocampal dysplasia and axonal degeneration	Tau‐specific pathology has not been reported
	Ataxia telangiectasia	Atm^R35X/R35X^/ Aptx^−/−^ mice (Perez et al., 2021)	Progressively severe ataxic phenotype Perturbed cerebellar Purkinje neurons	Double mutation adds another layer of complexity Tau‐specific pathology has not been reported
	Werner syndrome	Wrn^Δhel/Δhel^ mice (Hui et al., 2018)	Changes in cognitive, locomotor and social behaviour	No change in novelty memory Tau‐specific pathology has not been reported
	Xeroderma pigmentosum	Subtype‐dependent (see Table 4)		

### 2.2 Transgenic progeroid model type 1: laminopathy models

Laminopathies are a diverse group of diseases caused by genetic malfunctions of proteins associated with the nuclear lamina—a protein meshwork coating the inner surface of the nuclear envelope and forming a part of the nucleoskeleton (Shin & Worman, 2021). The nuclear lamina is critical for maintaining the structure of the nucleus and the organisation of the chromatin. As the most common form of laminopathy, Hutchinson Gilford progeria syndrome (HGPS) is caused by an autosomal dominant mutation in *LMNA*, the gene encoding for lamin A and C proteins. The mutation leads to the production of a truncated form of lamin A (termed ‘progerin’; Ullrich & Gordon, 2015). Healthy lamin A precursor undergoes a 4‐step post‐translational process to produce mature lamin A: normal wild‐type pre‐lamin A undergoes farnesylation at its C‐terminal CaaX box by farnesyltransferase (FTase), C‐terminal cleavage after the cysteine residue, methylation by protein‐S‐isoprenylcysteine carboxyl methyltransferase and upstream cleavage by ZMPSTE24 (Davies et al., 2009). In HGPS, due to its permanent farnesylation state, progerin is constitutively retained in the nuclear lamina, acting as a dominant negative and leading to multiple defects relevant to cellular ageing. These include altered mechanical properties of the nuclei and increased nuclear stiffness (Goldman et al., 2004), altered chromatin organisation (Shevelyov & Ulianov, 2019), increased unrepaired DNA double‐stranded breaks (Zhang et al., 2016), disrupted nucleocytoplasmic transport (Kelley et al., 2011; Snow et al., 2013) and increased protein translation rate (Buchwalter & Hetzer, 2017). Models that recapitulate both tauopathy and laminopathy features have been built upon mutations in *LMNA*.

The first attempt of using progerin to accelerate ageing in a mouse model was accomplished in 2012. In knock‐in mice that express exclusively lamin A or exclusively progerin, it became apparent that lamin A, prelamin A and progerin are absent in neuronal and glial cells, suggesting that lamin A and its derivatives are not likely implicated in brain ageing (Jung et al., 2012). A neural‐specific microRNA, miR9, has been identified to suppress lamin A—and therefore progerin expression in the central nervous system; this finding was confirmed by an independent study performed on induced pluripotent stem cells derived from HGPS patient cells (Nissan et al., 2012). This is in agreement with the absence of Alzheimer's‐like pathology in HGPS post‐mortem tissues and with the clinical observations that HGPS patients do not develop neurological symptoms such as cognitive deterioration (Ullrich & Gordon, 2015). In Baek et al., 2015, the authors established an inducible HGPS transgenic mouse model that specifically expresses progerin in bone, skin, heart and neurons. This system showed that forced long‐term expression progerin in the hippocampal neurons resulted in severe nuclear distortions (e.g., nuclear blebbing and invagination) in cells from all tissues, without any apparent neuropathological defects, such as protein aggregation, adult neurogenesis ability, transcriptomic profile alteration (including lamin B1) and behavioural changes (Baek et al., 2015). It therefore suggests that progerin‐induced nuclear dysmorphology in neurons does not necessarily correlate with loss of neuronal functions and dementia‐related pathologies. The mechanisms behind this neuronal protection remain unknown. It is worth mentioning that progerin has also been used to assist in modelling pathological phenotypes of late‐onset Parkinson's disease in human iPSC‐derived neurons (Miller et al., 2013), where expression of progerin in PD iPSC‐derived dopamine neurons induced ageing‐related markers and disease phenotypes, such as dendrite degeneration, Lewy‐body‐precursor inclusions and dysregulated protein degradation mechanisms.

Recently, the role of the nuclear lamina in Alzheimer's disease has attracted more attention (Gil et al., 2021). Analyses of AD post‐mortem brain tissue revealed that lamin A mRNA levels increased at the late stage of AD in the hippocampus (Méndez‐López et al., 2019). Immunohistochemistry analysis further demonstrated the presence of lamin A immunopositive pyramidal neurons in the CA1 and CA3 regions of the hippocampus in AD brains from the Braak stages I to VI (Gil et al., 2020). Given these discoveries, the notion that lamin A and its derivatives are absent in neuronal cells and are therefore not involved in the function of the central nervous system deserves renewed scrutiny.

Apart from expressing progerin, ZMPSTE24, as an enzyme essential in lamin A processing, has also been exploited to generate progeroid ageing features in experimental model systems. ZMPSTE24 is an integral membrane zinc metallopeptidase that endoproteolytically cleaves the C‐terminal region of carboxymethylated prelamin A and produces mature lamin A (Quigley et al., 2013). Twenty human ZMPSTE24 mutations that reduce the enzyme activity have been identified to associate with three disease categories of increasing severity: mandibuloacral dysplasia type B, severe progeria (atypical ‘HGPS’) and restrictive dermopathy (Barrowman et al., 2012); the crucial involvement of ZMPSTE24 in laminopathies was further emphasised by a novel mouse model expressing non‐cleavable prelamin A (Wang, Shilagardi, et al., 2022). Despite its availability (Bergo et al., 2002; Varela et al., 2005), to the best of our knowledge, ZMPSTE24‐deficient mice have not been directly used to study brain ageing in neurodegenerative diseases. Nevertheless, neurological abnormalities, such as microcephalia (de Carlos et al., 2008) and oesophageal achalasia (Yang et al., 2015), have been observed in ZMPSTE24‐deficient mice, suggesting potential functions of ZMPSTE24 and lamin A in the nervous tissues.

It is noteworthy that multiple recent evidence has highlighted the involvement of other nuclear lamina proteins in tauopathies. In a TgTau Drosophila model, it has been demonstrated that a pathogenic mutation of tau (R406W) alters the arrangement of B‐type lamins, affecting maintenance of genomic architecture, cell cycle regulation and survival of adult neurons (Frost et al., 2016). Analysis of post‐mortem human FTLD‐MAPT cortex revealed a high incidence of nuclear deformation, indicating that tau mediates nuclear membrane dysfunction (Paonessa et al., 2019). Interestingly, two independent tissue culture‐based CRISPRi screening both pointed out potential involvement of BANF1 (Barrier‐to‐autointegration factor 1) in tau aggregation (Koss et al., 2022; Polanco et al., 2022). As loss of BANF1 has significant implications in nuclear envelope integrity in Nestor–Guillermo progeria syndrome (NGPS; Janssen et al., 2022), these findings raised the possibility of encompassing NGPS into the laminopathy‐tauopathy experimental model repertoire.

### 2.3 Transgenic progeroid model type 2: DNA repair deficiency models

DNA damage is a feature of both age‐related neurodegenerative diseases and of PS. Accumulation of DNA damage is a cardinal factor in physiological ageing as it can lead to cell death, senescence, stem cell loss and polyploidisation (reviewed by Schumacher et al., 2021). Unsurprisingly, reduced genomic stability has been a widely reported feature in Alzheimer's disease (reviewed by Lin et al., 2020). Immunostaining for γH2AX, a phosphorylated form of H2AX that is widely used as a marker of double‐stranded DNA breaks (DSBs), showed that DSBs accumulate in neurons and astrocytes in the hippocampus and frontal cortex of AD patients during the progression of neurodegeneration (Shanbhag et al., 2019). Consistent with these observations, in vitro studies with primary mouse cortical neurons revealed that non‐phosphorylated tau accumulates perinuclearly upon DSBs formation, followed by accumulation of phosphorylated tau immunoreactive to AT8 antibody (Asada‐Utsugi et al., 2022). In the field of pathological ageing, deficiencies in the DNA repair machineries result in many PS such as Werner syndrome (Muftuoglu et al., 2008), Cockayne syndrome (Laugel, 2013) and xeroderma pigmentosum (Lehmann et al., 2011). The wealth of DNA repair‐deficient models established for studying these progeroid syndromes offered an opportunity to explore neurodegeneration in a high DNA damage susceptibility system.

Werner syndrome (WS) is a DNA repair‐related premature ageing syndrome caused by mutations in a RecQ family DNA helicase, WRN (Muftuoglu et al., 2008). In contrast to progeroid laminopathies, Werner syndrome has a delayed onset—typically recognised by the third or fourth decades of life and is therefore sometimes referred to as ‘adult progeria’ (Oshima et al., 2017). Thus far, neurodegeneration has not been observed in individuals with WS. Nonetheless, though rare, non‐AD senile dementia, schizophrenia (Goto, 1997), cerebrovascular disease and peripheral neuropathy (Anderson & Haas, 2003) have been reported as WS‐associated neurological complications. The rarity of neurological manifestation in the central nervous system discouraged the attempts of accelerating neuronal ageing with *WRN* mutations. However, a recent longitudinal behavioural assessment on transgenic mice bearing a WRN helicase deletion (Wrn^Δhel/Δhel^) demonstrated a loss of motor activity and coordination, reduction in perception, increase in repetitive behaviour and deficits in both spatial and social novelty memories in *WRN* mutant mice compared to age‐matched wild‐type mice, possibly through microglial dysfunction and elevated neuronal oxidative stress (Hui et al., 2018). Although this finding may benefit from wider validation in other model organisms, it encourages the use of WRN mutation in neurological models.

Xeroderma pigmentosum (XP) is a recessively inherited rare skin disorder caused by defects in enzymes responsible for nucleotide excision repair (Lehmann et al., 2011), leading to high vulnerability to UV‐induced damage, hypersensitivity to sunlight and high susceptibility to skin cancer. There are nine different genetic subtypes of XP depending on the affected gene: XPA, XPB (or ERCC3), XPC, XPD (or ERCC2), XPE (or DDB2), XPF (or ERCC4), XPG (or ERCC5), XPV (or POLH) and ERCC1 (Kraemer et al., 2003). Depending on the disease subtype, XP patients may have different neurological manifestations. Of note, cognitive dysfunction may develop in childhood and advance through later stages in XPA, and peripheral neuropathy has been reported in XPG patients (Anttinen et al., 2008). Table 4 summarises mouse models established for each XP subtype. In contrast to the prominent neurological phenotypes in XPA patients, Xpa^−/−^ mice only displayed mild neurological manifestations, such as delayed neuromotor recovery and increased memory acquisition dysfunction following experimental brain trauma (Tomasevic et al., 2012). Interestingly, overt neuropathology and cognitive/behavioural alterations have been observed in transgenic mouse models for XPG and ERCC1 (Barnhoorn et al., 2014; Borgesius et al., 2011; de Waard et al., 2010; Lawrence et al., 2008). Despite that no protein misfolding‐related pathology had been reported in XP patients or in these transgenic XP mouse models, the neurodegenerative features observed in XPG and ERCC1 models indicate that defects in nucleotide excision repair mechanism alone may lead to neurodegeneration and conformational assessment of aggregation‐prone proteins (e.g., tau, Aβ and α‐synuclein) may be a useful future step in XPG‐ and ERCC1‐deficient mouse models.

**TABLE 4:** Summary of mouse models of xeroderma pigmentosum.

XP subtype	Mouse model	Neuropathology in brain	Neurological manifestation
XPA	Xpa^−/−^	No (de Vries et al., 1995; Nakane et al., 1995)	Delayed neuromotor recovery and increased memory acquisition dysfunction following experimental brain trauma (Tomasevic et al., 2012)
XPB	Xpb^XPCS^ ^a^	No (Andressoo et al., 2009)	No (Andressoo et al., 2009)
	Xpb^y/y^	Unknown (Donnio et al., 2019)	Unknown
XPC	Xpc^−/−^	Unknown (Melis et al., 2013)	Unknown
XPD	Xpd^R722W^	Unknown (de Boer et al., 1998)	Unknown
	Xpd^XPCS^ ^a^	No	Spastic and abnormal coordination of hindlimbs in male animals (Andressoo et al., 2006)
XPE	Ddb2^−/−^	Unknown (Yoon et al., 2005)	Unknown
XPF	Ercc4^em1(IMPC)J^	Unknown	Unknown (https://www.jax.org/strain/033920)
XPG	Xpg^−/−^	Age‐related accumulation of neurodegenerative changes in central nervous system: prominent astrocytosis, loss of Purkinje cells and increased apoptosis in the cerebrum and the cerebellum at 14 weeks Smaller neocortex Ventricle enlargement (Barnhoorn et al., 2014)	Gait ataxia Action tremor Cognitive decline
XPV	Polh^−/−^	Unknown (Martomo et al., 2005)	Unknown
ERCC1	Ercc1^Δ/−^	Age‐related motor neuron degeneration: widespread astrocytosis and microgliosis, and motor neuron loss and denervation of skeletal muscle fibres (de Waard et al., 2010) Age‐related neurodegeneration: reactive astrocytosis, mild neuronal degeneration, signs of genotoxic stress and reduced hippocampal synaptic plasticity (Borgesius et al., 2011) Mild neurodegenerative changes: peripheral (sciatic) nerve vacuolisation, brain mass reduction (Dollé et al., 2011)	Progressive motor abnormalities and reduced life span: clasping of the hindlimbs, fine tremors and kyphosis, severe locomotor deficits and reduced ability to maintain balance (de Waard et al., 2010) Impaired fear conditioning and impaired water maze performance at 6 months of age (Borgesius et al., 2011)
	Ercc1^−/−^	No histopathological neurodegeneration, or of abnormal neuromuscular junctions. Observed uraemic encephalopathy (Lawrence et al., 2008)	Poor coordination, ataxia and loss of visual acuity (Lawrence et al., 2008)

^a^ Double transgenic models that also include Cockayne syndrome‐related mutations.

Cockayne syndrome (CS) is a rare, autosomal recessively inherited genetic disorder characterised by premature ageing‐like features, such as severe growth failure and cutaneous photosensitivity. Defects of two genes, CSA (ERCC8) and CSB (ERCC6), are responsible for the disease (Henning et al., 1995; Troelstra et al., 1992). In contrast to other progeroid syndromes associated with DNA repair deficiencies, CS is a degenerative disorder and has more profound manifestations in the central nervous system, including mental retardation, microcephaly, retinal atrophy and progressive neurodegeneration (Spitz et al., 2021). To study neurological impairments caused by CSA and CSB deficiency, multiple animal models have been established. Knocking out CSA (Csa^−/−^) in mice showed no detectable neurodegeneration at 26 weeks of age; however, it revealed that CSA deficiency leads to increase of p53‐positive neurons in neocortex, cerebellar cortex and spinal cord (Jaarsma et al., 2011). A double mutant mouse model (Csa^−/−^/Xpa^−/−^) presents neurological dysfunction resembling CS patients, including myelin loss and loss of Purkinje cells. In animal models of CSB, microglia activation and astrocytosis have been reported in white matter regions in Csb^−/−^ mice (Jaarsma et al., 2011). Similarly, using CSB‐deficient rats (Csb^R571X/R571X^), Xu et al. observed multiple neurological defects, including cerebellar atrophy, hippocampal dysplasia, axonal degeneration and astrocyte activation in cerebella (Xu et al., 2019). Beyond rodent models, Csb‐1 deficiency in *C. elegans* also associates with locomotion dysfunction and aged‐dependent loss of sensitivity to mechanosensory stimuli, possibly through its effects on mitochondrial activity (Lopes et al., 2020). It is noteworthy that protein misfolding and loss of proteostasis have been reported in CS patient‐derived fibroblasts (Alupei et al., 2018; Qiang et al., 2021). However, it is currently unclear if these proteostatic defects can be mirrored in CSA or CSB animal models, and whether this may accelerate the progression of proteinopathies in the central nervous system, such as tau aggregation.

Ataxia telangiectasia (A‐T) is a neuromotor dysfunction neurodegenerative disorder of childhood caused by the disruption of gene ATM (Ataxia‐telangiectasia mutated serine/threonine kinase). Clinically, A‐T patients represent a wide variety of symptoms including progressive cerebellar ataxia, oculocutaneous telangiectasia, variable immunodeficiency, radiosensitivity, susceptibility to malignancies and increased metabolic diseases (Amirifar et al., 2019). In an ATM knockout Drosophila model, it has been demonstrated that reducing the function of ATM significantly enhances tau‐induced neuronal apoptosis and exacerbates tau neurotoxicity (Khurana et al., 2012). To further increase the genotoxic stress, a novel double mutant mouse model, Atm^R35X/R35X^/Aptx^−/−^, has been generated; it not only develops a progressively severe ataxic phenotype but also exhibits significantly perturbed cerebellar Purkinje neurons (Perez et al., 2021). Of note, examination of Alzheimer's disease post‐mortem brain samples revealed an elevated level of ATM compared to the age‐matched control (Katsel et al., 2013), suggesting that activation of the DNA damage checkpoint is a shared feature between A‐T and AD.

### 2.4 Non‐progeroid models of accelerated ageing

Apart from the aforementioned transgenic animal models where the progeric alleles are artificially introduced to the genome to mimic ageing, there are other models to study accelerated ageing of the brain. Here, we briefly review Klotho‐deficient mice, SAMP8 mice and African Turquoise Killifish.

Klotho‐deficient (KL^−/−^) mice are a widely used animal model in ageing research. Named after one of the three Fates in Greek mythology, Klotho was discovered fortuitously as a monogenic recessive trait by Kuro‐o et al. in Kuro‐o et al., 1997, triggered by a transgene insertional inactivation event (Kuro‐o et al., 1997). There are three subfamilies of Klotho, α‐, β‐ and γ‐Klotho, and they have different physiological functions (Dolegowska et al., 2019). In the context of ageing biology, the term ‘Klotho’ generally refers to the α‐Klotho subfamily. Encoded by the KL gene, α‐Klotho has multiple molecular functions (e.g., β‐glucosidase activity and fibroblast growth factor receptor binding activity [Gaudet et al., 2011]) and has a wide variety of cellular implications (e.g., mitochondrial dysfunction [Sahu et al., 2018]). In the context of neurodegenerative disorders, the level of Klotho in the central nervous system is generally negatively correlated with the severity of proteinopathies. For instance, a longitudinal study in 2020 suggested that increased serum level of Klotho (in Klotho‐VS heterozygous individuals) is associated with reduced AD risk in APOE4 carriers from 60 to 80 years of age (Belloy et al., 2020). It was later reported that Klotho‐VS heterozygosity was associated with a lower cross‐sectional and longitudinal increase in amyloid‐related tau pathology and tau‐related memory defects (Neitzel et al., 2021). Echoed with these findings were two recent clinic observations suggesting that higher levels of CSF Klotho were associated with lower CSF Aβ42 and tau burden (total tau and phosphorylated tau; Driscoll et al., 2021; Grøntvedt et al., 2022). In the experimental realm, Klotho‐deficient mice display overt phenotypes compared to their wild‐type littermates, such as hypogonadism, premature thymic involution, ectopic calcification, impaired bone mineralisation, skin atrophy, hearing loss and neurodegeneration (Kuro‐o, 2009). Using a KL^−/−^ mouse model, Nagai et al. showed that deficiency of Klotho in mouse brain results in an impairment of visual recognition memory and associative fear memory, which may be explained by an elevated level of apoptosis and oxidative stress in hippocampus (Nagai et al., 2003). More recently, Leon et al. showed that peripherally administered Klotho fragment (αKL‐F) can enhance cognitive parameters and neural resilience in transgenic α‐synuclein mice (Leon et al., 2017). Dubal et al., using hAPP (human amyloid beta precursor protein) transgenic mice that had been crossed with Klotho transgenic mice, demonstrated that elevated level of Klotho protects hAPP mice against premature mortality and cognitive impairments (Dubal et al., 2015). These studies altogether suggest a neural‐protective role of Klotho and this mechanism deserves further investigation in simplified tau aggregation reporter platforms, such as in the HEK293 tauRD‐YFP cell line (Sanders et al., 2014), and animal models carrying pathogenic tau mutations.

In pursuit of polygenic traits, senescence‐accelerated mouse prone (SAMP) substrains were established by Kyoto University through phenotypic selection from AKR/J breeding colonies. SAMP8 is one of the nine major SAMP substrains and shows features of rapid ageing. Functionally, SAMP8 mice show impairment of memory, deteriorations in learning ability (Miyamoto et al., 1986), emotional disorders (Miyamoto et al., 1992), altered circadian cycle and water consumption (Miyamoto, 1997). Pathologically, brain stem spongy degeneration, blood–brain barrier dysfunction and loss of cholinergic neurons are major anatomical changes in the central nervous system. Despite the absence of amyloid plaques and neurofibrillary tangles, SAMP8 mice show many cellular and molecular characteristics that mimic physiological brain ageing, such as increase in phosphorylated tau and increase in oxidative stress, severe cellular senescence, downregulation of glucose metabolism (Akiguchi et al., 2017) and age‐dependent neuroinflammations (Fernández et al., 2021). Moreover, amyloid‐β granules are present in the stratum radiatum of the CA1 region of hippocampus in SAMP8 mice, which contain Aβ42, Aβ40, tau, MAP2 and α‐synuclein peptides (Manich et al., 2011). An attempt to combine the characteristics of the APP/PS1 transgenic mouse model with a senescence‐accelerated background of SAMP8 mice revealed cognitive abnormalities, amyloid plaque formation and other AD markers (e.g., neuroinflammation, hyperphosphorylation of tau), but not neurofibrillary tangles (Porquet et al., 2015). To the best of our knowledge, a SAMP8‐based mouse model that expresses human tau has not been established but it may be a useful model.

African Turquoise Killifish (*Nothobranchius furzeri*) is a species of small freshwater fish native to southeast Africa, primarily found in Zimbabwe and Mozambique. Due to its small size and rapid life cycle, this fish has recently gained a resurgence of interest as an experimental model system in ageing science (Smith et al., 2017). Compared to other species, this species of fish reaches sexual maturity in 3–4 weeks (Terzibasi et al., 2008) and has a very short lifespan (9–16 weeks for the GRZ strain, and 23–28 weeks for the MZM‐0403 strain [Terzibasi et al., 2008]), 6–10 times shorter than that of zebrafish (Kishi et al., 2009). Their small size, fast reproductive cycle and easy‐to‐care‐for nature also make them an ideal species for laboratory experiments. Furthermore, the African Turquoise Killifish genome contains 96.8% of core eukaryotic genes and has been well annotated (Valenzano et al., 2015), thus making it a suitable vertebrate model organism for the study of ageing at an organismal level. In a preprint that recently became available on bioRxiv, using African Turquoise Killifish, Harel et al. identified that DDX5 (ATP‐dependent RNA helicase DDX5), a prion‐like RNA binding protein, forms mislocalised cytoplasmic aggregates in the brains of aged killifish (7 months) and such aggregates can be propagated in DDX5 aggregation reporter yeast (Harel et al., 2022), suggesting that transmissible prion‐like protein aggregates accumulate in the brain during vertebrate ageing. Despite that misfolded tau was not observed in protein aggregates that accumulate in old killifish brains, this pioneering observation encouraged more thorough examination of aged African Turquoise Killifish for tau‐specific pathologies. Indeed, (a) DDX5 is directly involved in exon 10 splicing of the MAPT gene (Kar et al., 2011); (b) similar to zebrafish (MacRae & Peterson, 2015), rapid reproduction and small size of African Turquoise Killifish allows it to be used in phenotype‐based screening in drug discovery; (c) existing protocols for genome engineering (Harel et al., 2016; Hartmann & Englert, 2012) allows for establishment of transgenic killifish expressing human MAPT gene.

### 2.5 Slow ageing model

#### 2.5.1 Naked mole rat

The naked mole‐rat (NMR; Heterocephalus glaber) has attracted considerable biogerontological interest for multiple reasons. First, they have extraordinary longevity. As the longest‐lived rodent, the NMR has a maximal lifespan of more than 30 years in a laboratory environment (Ruby et al., 2018), 10‐fold greater than the allometrically predicted figure for a mouse‐sized rodent (Edrey et al., 2011). Second, the NMR has a high tolerance to stress, such as hypoxia (Park et al., 2017; Xiao et al., 2017) and hypercapnia (Clayson et al., 2020). Third, the NMR possesses unusual resistance against diseases. For instance, the NMR has a low susceptibility to spontaneous cancer and experimentally induced tumour growth (Liang et al., 2010), yet the mechanism behind this observation is currently controversial (Hadi et al., 2020; Tian et al., 2013). In the context of neurodegenerative diseases, low susceptibility of protein aggregation for tau and amyloid‐β has been observed in the central nervous system of naked mole rat (Edrey et al., 2013; Orr et al., 2015). NMRs offer a unique slow‐ageing system to study modulators of tau aggregation due to high sequence similarity between human tau and NMR tau—protein alignment comparison illustrates 95% overall identify and 100% identity at the functional microtubule‐binding domain (Orr et al., 2015). Unlike what is observed in human, NMR tau undergoes a progressive increase in molecular weight during development, a transition from a 62/72 kDa doublet to a 72 kDa singlet at 3 weeks of age, and to an 88 kDa band later (Orr et al., 2015). Interestingly, despite that NMRs have a considerable level of total tau and phospho‐tau, NMR tau remains localised in the axon and does not cause pathologies (Orr et al., 2015). It remains unclear whether NMR tau undergoes aggregation, and whether NMRs possess species‐specific mechanisms that might prevent pathological tau deposition. Thus far, a very limited number of studies have been conducted on NMR tau; however, understanding the post‐translational modifications of NMR tau and determining its aggregation propensity in both in vitro and in vivo experiments will extend our understanding of potential NMR‐specific mechanisms that could prevent tau misfolding.

#### 2.5.2 Assessing the biological age in animal models of tauopathies

If using accelerated ageing models to study age‐related diseases such as tauopathies, it would be desirable to be able to measure the biological age of the animal models being used. Unlike chronological age, which is based solely on an organism's date of birth, biological age is instead a measure of an individual's age‐related risk of adverse outcomes. For humans, several biomarkers have been proposed which can be used to assess biological age, including telomere length, metabolic age scores and composite biomarkers (i.e., the combination of several biomarkers; Jylhävä et al., 2017). However, the translation of these biomarkers to non‐human animal models of ageing is complex. Taking telomere lengths as an example, initial telomere length and the rate of telomere shortening can vary substantially between species (Calado & Dumitriu, 2013). On the contrary, the ‘epigenetic clocks’ which predict an organism's biological age based upon DNA methylation at specific CpG sites have shown promise in being applied to other species. First developed as a predictor of biological age in humans (Bocklandt et al., 2011), this approach has been well validated in predicting biological age in many other species including mice (Stubbs et al., 2017), rats (Levine et al., 2020), great apes (Horvath et al., 2021) and ruminants (Caulton et al., 2021) to name a few.

Importantly, epigenetic clocks have also been developed for specific tissues, including brain. Biological age has been shown to correlate with tau load in the normal human brain (Maroni et al., 2020), and with risk of dementia in humans (Levine et al., 2015; Wu et al., 2021), although some studies have cast doubt on this association (Zhou et al., 2022). Given the inherent discrepancy between chronological and biological age in accelerated ageing models, being able to determine their biological age would be useful for a proper interpretation of the results. In mouse brain tissue, for which a specific epigenetic ageing clock has been developed (Coninx et al., 2020), the triple transgenic Alzheimer's Disease (3xTg‐AD) mouse model showed increased biological age relative to chronological age‐matched brain controls. Thus, these methylation‐based ‘epigenetic clocks’ might be a useful tool to measuring the biological age of tissues including brain in accelerated ageing animal models of dementia.

#### 2.5.3 Outlook: Searching for the molecular origin of tau misfolding using animal models

In recent years, animal models with experimentally induced ageing features have emerged as a promising avenue for exploring sporadic tauopathies and other age‐dependent neurodegenerative diseases. This expanding interest has been supported by the rapidly improving gene editing technologies, extensive experience in husbandry and high‐quality genomic and transcriptomic sequencing data. Yet, despite increasing research on the determinants of tau aggregate formation, it is still not clear which molecular mechanisms drive the production of abnormal tau in idiopathic tauopathies. Thus far, most efforts have been put into in vitro experiments where tau aggregation is primarily driven by the presence of polyanionic inducers (Ingham et al., 2022) or specific combinations of salt ingredients (Lövestam et al., 2022)—the physiological relevance of these findings will need to be validated in human patient samples and in animal models. To the best of our knowledge, despite that many ageing model organisms have neurological manifestations, such as microgliosis in ERCC1‐deficient mice (de Waard et al., 2010) and amyloid‐β granules in SAMP8 mice (Manich et al., 2011), none of these canonical models displays spontaneous accumulation of tau in the central nervous system. It follows that acceleration of ageing driven by progeric mutations or polygenic traits have not yet recapitulated an environment that accurately mimics an aged brain. This obstacle thus begs the question ‘is it possible to establish alternative models to study delayed and accelerated brain ageing?’ Indeed, non‐canonical ageing model organisms have been elegantly summarised by Holtze et al., 2021. The wide range of lifespan of these models (ranging from <1 month for *C. elegans* to potential immortality for *S. mediterranea*) may help gain a more holistic understanding of the ageing process in the nervous tissues. It should be noted that tau misfolding may give rise to structurally distinct pathogenic species in different tauopathies (reviewed by [Han et al., 2022]). A future model should thus be expected to exhibit heterogeneity of tau conformers at an advanced biological age, similar to a low‐tau expression transgenic mouse model previously reported (Daude et al., 2020; Eskandari‐Sedighi et al., 2017). Apart from these animal models, certain experimental manipulations have also been employed to investigate the effects of ageing on neurodegeneration, such as parabiosis (a specialised type of blood transfusion procedure that surgically combines young and aged animals to study their physiological interactions [Hu et al., 2022; Kim et al., 2020; Wang et al., 2018; Zhao et al., 2020]) and caloric restriction (Brownlow et al., 2014; Cox et al., 2019; Müller et al., 2021). While current model organisms with ageing features have not yet fully recapitulated the physiological features associated with sporadic tauopathies, the early molecular events at the headwater of idiopathic proteinopathies may become better elucidated by more thorough characterisation of the cellular environment in naturally aged brain and combined use of transgenic animal models with age‐related experimental manipulations.

## AUTHORS' CONTRIBUTIONS

Z.Z.H. and A.F. wrote the original draft of the article with insights from D.L. D.L. reviewed and edited the article.

## FUNDING INFORMATION

D.L. was supported by a Sir Henry Dale Fellowship jointly funded by the Wellcome Trust and the Royal Society (Grant Number 206242/Z/17/Z). Z.Z.H. was supported by a Cambridge Trust Cambridge International Scholarship. A.F. was supported by funding from the International Journal of Experimental Pathology.

## CONFLICT OF INTEREST STATEMENT

D. L. is a co‐founder of Adrestia Therapeutics and a scientific advisor for Shift Bioscience and Adrestia Therapeutics. The other authors declare that they have no conflicts of interest.

## Data Availability

Not applicable.
